# Bimaxillary Orthognathic Surgery for Facial Asymmetry with Near-Normal Sagittal Relationship: Mid-Term Stability and Remodeling

**DOI:** 10.3390/medicina62020372

**Published:** 2026-02-13

**Authors:** Yuhung Lin, Chenyu Liao, Yunfeng Li

**Affiliations:** State Key Laboratory of Oral Diseases & National Center for Stomatology & National Clinical Research Center for Oral Diseases & Department of Oral and Maxillofacial Surgery, West China Hospital of Stomatology, Sichuan University, Chengdu 610041, China; 2022224030038@stu.scu.edu.cn (Y.L.); liaochenyu@alu.scu.edu.cn (C.L.)

**Keywords:** bimaxillary orthognathic surgery, facial asymmetry, postoperative stability, skeletal adaptation

## Abstract

*Background and Objectives*: Mid-term skeletal stability after bimaxillary orthognathic surgery in patients with facial asymmetry and a relatively normal sagittal skeletal relationship (ANB ≈ 1–4°) remains underreported. This study aimed to determine the three-dimensional characteristics and temporal changes in postoperative skeletal remodeling and symmetry maintenance in such patients. *Materials and Methods*: This retrospective case series included 25 patients (ANB ≈ 1–4°) undergoing bimaxillary orthognathic surgery. Three-dimensional computed tomography was performed preoperatively (T0), immediately postoperatively (T1), and at 6–12 months postoperatively (T2) to quantify bilateral condylar, ramus, mandibular body, maxillary parameters, and occlusal cant. Statistical analyses were performed using appropriate statistical methods for paired and repeated-measures designs. *Results*: Preoperatively, the long side exhibited significantly greater condylar volume, ramus height, and mandibular body length than the short side (all *p* < 0.05). Postoperatively, a “long-side reduction and short-side augmentation” strategy significantly reduced or reversed most bilateral differences, with a marked improvement in occlusal plane cant (*p* < 0.01). At T2, only mild bone remodeling was observed, with no significant loss of postoperative skeletal symmetry. The occlusal plane remained stable. *Conclusions*: In patients without marked sagittal discrepancies, bimaxillary orthognathic surgery effectively restores transverse and vertical skeletal symmetry. Mid-term stability is well maintained over 6–12 months, with only mild condylar and ramus remodeling, suggesting adaptive remodeling rather than relapse.

## 1. Introduction

Facial asymmetry is a frequent clinical manifestation among individuals with dentofacial deformities. Epidemiological studies have indicated that approximately one-third of patients undergoing orthognathic surgery present with clinically discernible facial asymmetry, with chin deviation being the most prevalent feature. Vertical asymmetries, such as occlusal plane cant, are also frequently observed in these patients [1,2,3]. In the present study, the term facial asymmetry with a relatively normal sagittal skeletal relationship (ANB ≈ 1–4°) denotes cases characterized by marked mandibular deviation or facial asymmetry, while maintaining a near-normal maxillomandibular sagittal relationship and lacking pronounced mandibular prognathism, retrognathism, anterior crossbite, or deep overbite [2]. Although the occlusal relationship in these patients superficially resembles that of Class I malocclusion, substantial skeletal asymmetry exists in the transverse or vertical plane [4], posing significant challenges for diagnosis and surgical planning, and increasing the risk of misinterpretation as a purely dentoalveolar problem [5].

The mandibular condyle is widely recognized as a critical determinant influencing the outcomes of orthognathic surgery [6]. As an integral component of the temporomandibular joint, it plays an essential role in mandibular growth, functional movements, and the postoperative maintenance of skeletal stability and symmetry [6]. Evidence from previous studies indicates that condylar resorption or asymmetry can contribute to skeletal relapse, occlusal instability, and temporomandibular joint dysfunction following orthognathic procedures [7]. Accordingly, comprehensive preoperative imaging evaluation of the condyle and long-term postoperative monitoring are considered crucial for optimizing surgical outcomes [7,8].

While postoperative three-dimensional (3D) skeletal changes in patients with skeletal Class III malocclusion combined with facial asymmetry have been extensively investigated in recent years [9,10], evidence concerning patients with facial asymmetry but a relatively normal sagittal skeletal relationship remains scarce and insufficiently elucidated [11]. Given that the anteroposterior skeletal relationship in this subgroup approximates normal, treatment is predominantly directed toward structural corrections in the transverse and vertical dimensions—a therapeutic paradigm that differs markedly from that employed in conventional skeletal Class III asymmetry. Bimaxillary orthognathic surgery necessitates concurrent remodeling of maxillary and mandibular spatial configurations, thereby compounding the complexity and unpredictability of postoperative skeletal stability [12,13]. To date, there is no consensus in the literature regarding postoperative symmetry maintenance, temporomandibular joint adaptation, or skeletal remodeling trajectories in this population, and existing studies remain largely exploratory [11,14].

To address these knowledge gaps, this study aimed to determine the three-dimensional characteristics and temporal changes in postoperative skeletal remodeling and symmetry maintenance among patients with facial asymmetry and a relatively normal sagittal skeletal relationship who underwent bimaxillary orthognathic surgery. Strict inclusion criteria were adopted to minimize confounding from sagittal discrepancies, restricting enrollment to patients with a near-normal maxillomandibular sagittal relationship (ANB ≈ 1–4°) and without evident prognathism or retrognathism. The findings are expected to clarify how maxillomandibular structural adaptation contributes to mid-term postoperative stability and to provide clinically relevant evidence for individualized postoperative evaluation and long-term stability prediction.

## 2. Materials and Methods

### 2.1. Study Population

A retrospective case series was conducted, enrolling 25 patients with facial asymmetry with a relatively normal sagittal skeletal relationship (ANB ≈ 1–4°) who underwent bimaxillary orthognathic surgery in the Department of Orthognathic and Temporomandibular Joint Surgery, West China Hospital of Stomatology, Sichuan University, between 2020 and 2024. The cohort comprised 8 males and 17 females, with a mean age of 24.4 ± 3.2 years. All patients had completed presurgical orthodontic treatment. To minimize the confounding influence of sagittal discrepancies such as prognathism or retrognathism, cases with marked sagittal abnormalities were strictly excluded. The study protocol was approved by the Ethics Committee of West China Hospital of Stomatology (approval No.: WCHSIRB-D-2025-309).

Eligibility for inclusion was determined according to the following conditions:

(1) A near-normal maxillomandibular sagittal relationship (ANB ≈ 1–4°) with evident mandibular deviation or facial asymmetry, defined as chin point deviation ≥ 4 mm from the facial midline, with or without dental midline shift or occlusal plane cant;
(2) Completion of skeletal growth (age ≥ 18 years) and absence of systemic diseases known to affect craniofacial development;
(3) No history of orthognathic surgery or severe maxillofacial trauma;
(4) Receipt of combined orthodontic–orthognathic treatment, with the surgical procedure comprising Le Fort I osteotomy of the maxilla combined with bilateral sagittal split ramus osteotomy (BSSRO) of the mandible, with or without genioplasty.

Exclusion from the study was determined according to the following conditions:

(1) Presence of pronounced skeletal Class II or Class III malocclusion;
(2) Coexisting congenital anomalies such as cleft lip/palate, jaw tumors, or osseous defects;
(3) Presence of temporomandibular joint pathology, including condylar constriction, arthritis, ankylosis, or other degenerative changes, as well as other conditions potentially affecting skeletal measurements, or severe systemic disorders.

### 2.2. Surgical Procedure

All patients in this study underwent bimaxillary orthognathic correction performed by a specialized orthognathic surgery team. The surgical protocol consisted of an intraoral Le Fort I maxillary osteotomy combined with bilateral sagittal split ramus osteotomy (BSSRO) of the mandible, enabling 3D repositioning of both jaws. Based on preoperative virtual surgical planning, the maxilla was elevated or horizontally shifted as necessary to correct occlusal plane cant and dental midline deviation. Following BSSRO, the distal segment of the mandible (tooth-bearing segment) was repositioned to the planned location, which involved correcting the deviated distal segment toward symmetry, with appropriate advancement or setback when indicated. In selected cases, genioplasty was performed to optimize chin symmetry.

Intermediate and final surgical splints, fabricated according to the virtual surgical planning, were used intraoperatively to guide accurate repositioning of the jaws and to establish the planned occlusal relationship. After repositioning, rigid internal fixation was achieved using titanium plates and screws to ensure postoperative stability of the bony segments. Particular attention was paid to the repositioning and maintenance of condylar position to avoid marked displacement or excessive stress. Small discontinuities of the anterior maxillary wall, often seen after Le Fort I osteotomy, were covered with autogenous cortical bone from the mandibular BSSRO segments when wide gaps were present. This adjunct restored continuity and healing without affecting skeletal stability. Postoperatively, all patients were fitted with customized occlusal splints and underwent intermaxillary fixation to facilitate bone healing and functional recovery.

### 2.3. Imaging and Measurement Protocol

**Figure 1:**
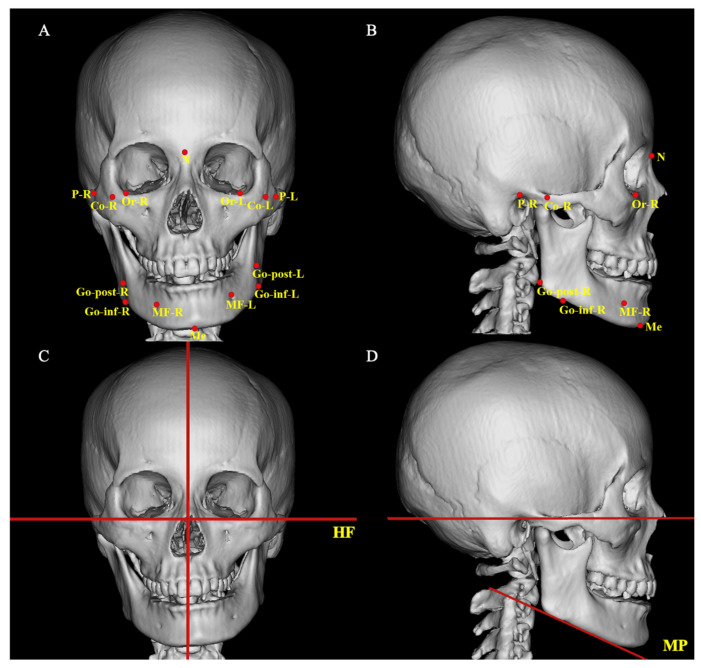
Anatomical landmarks (marked in red) and reference planes of the maxillofacial region. (**A**) Frontal view; (**B**) Lateral view; (**C**) Frontal view; (**D**) Lateral view.

All patients underwent cranial helical computed tomography (CT, Philips MX16 EVO, Aurora, IL, USA) scans at three time points: preoperatively (T0), approximately 2 weeks postoperatively (T1), and at least 6 months postoperatively (T2). Scans were acquired in the standard head position with the mandible in centric occlusion and at rest. The images were exported in DICOM format and imported into Mimics 21.0 software for 3D reconstruction. Following the 3D measurement methodology described by previous studies, a series of anatomical landmarks and reference planes were established on the reconstructed craniofacial skeletal models to assess morphological characteristics of the maxilla and mandible, as well as bilateral symmetry [15,16,17,18]. The side containing the Menton (Me) deviated from the facial midline was designated as the “short side,” and the contralateral side was designated as the “long side” for subsequent bilateral comparative analysis. The anatomical landmarks and reference planes used in this study are listed in Table 1, and the corresponding images illustrating the landmarks and planes are shown in Figure 1.

### 2.4. Measurement Parameters

**Figure 2:**
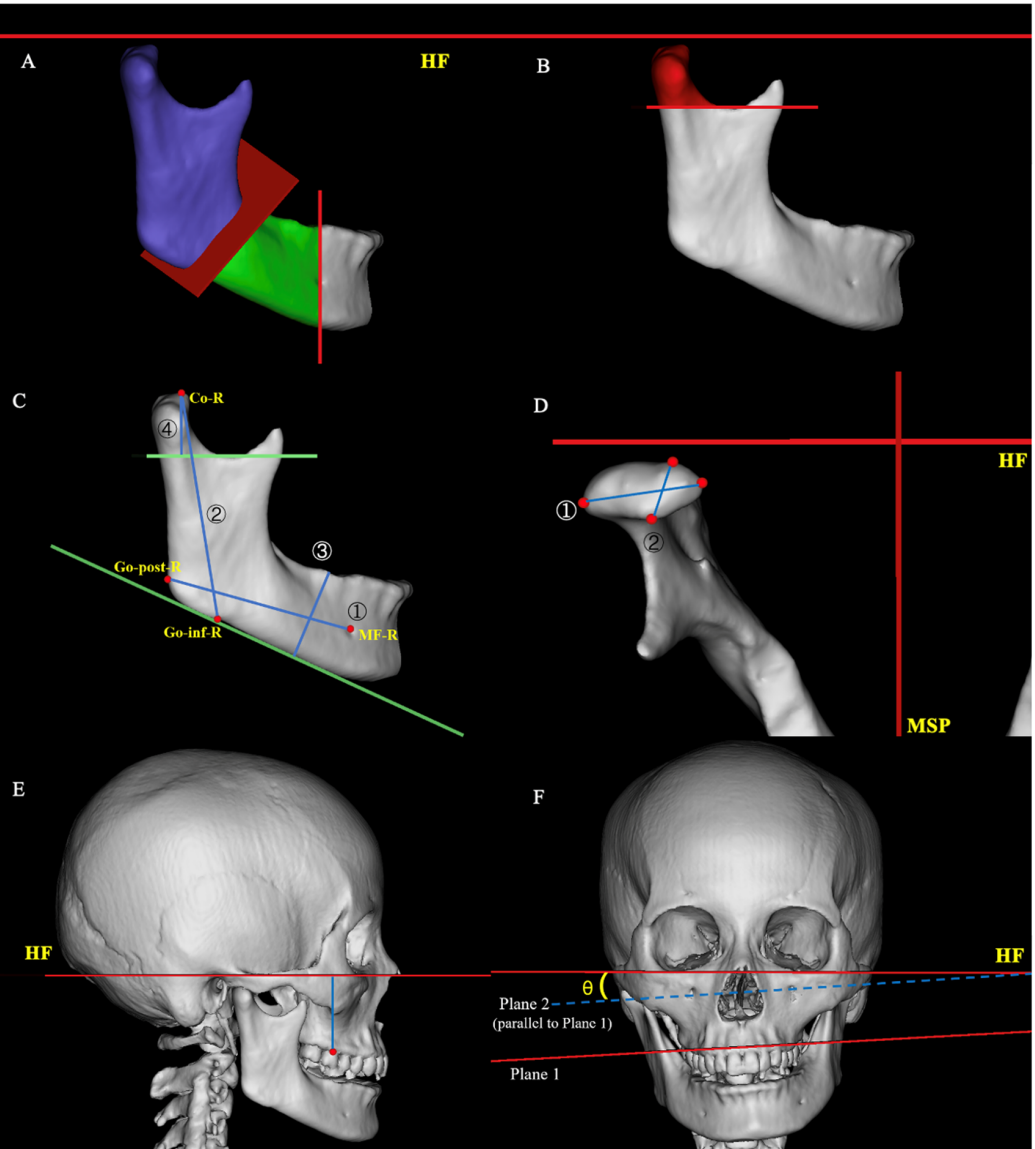
Three-dimensional measurement indicators and anatomical parameters. (**A**) Volumes of the mandibular ramus (purple) and mandibular body (green). (**B**) Volume of the condyle (red). (**C**) Anatomical measurements: ① mandibular body length, ② ramus height, ③ mandibular body height, ④ condylar height. (**D**) Condylar dimensions: ① mediolateral diameter, ② anteroposterior diameter. (**E**) Maxillary height (blue). (**F**) ∠θ: Occlusal cant angle.

Following the establishment of 3D anatomical landmarks and reference planes, all skeletal structural parameters were measured using Mimics software (Figure 2).

(1) Condylar volume (mm^3^): A cross-sectional plane parallel to the HF was established at the most inferior point of the sigmoid notch to isolate the condylar portion, and the enclosed 3D volume was calculated [19].
(2) Condylar dimensions (mm): The maximum transverse diameter (mediolateral width) was measured between the medial and lateral poles of the condyle, while the maximum anteroposterior diameter (anteroposterior length) was measured between the anterior and posterior poles [20].
(3) Ramus height (mm): The linear distance from the Co to the Go-inf was measured [17].
(4) Ramus volume (mm^3^): A reference plane was defined passing through the J-lat, J-med, and Go-inf to separate the mandibular ramus from the mandibular body, and the 3D volume of the ramus was calculated [20].
(5) Mandibular body length (mm): The linear distance from the Go-post to the MF was measured [20].
(6) Mandibular body height (mm): The perpendicular distance from the MP to the distal alveolar crest margin of the first molar was measured [20].
(7) Mandibular body volume (mm^3^): A sagittal plane parallel to the MSP was constructed through the MF to divide the mandible into the distal segment and the ramus segment; the volume of the distal segment (excluding the ramus) was then calculated. To avoid bias from concomitant genioplasty, the mental foramen plane perpendicular to the HF was used to separate the chin region, following a modified approach based on the previous study [20].
(8) Maxillary height (mm): The perpendicular distance from the most concave point of the buccal alveolar crest at the cervical margin of the first molar to the HF was measured [17].
(9) Occlusal cant angle (°): A plane (plane 1 in Figure 2F) was constructed through the most concave points of the buccal alveolar crest at the cervical margin of the bilateral maxillary first molars, oriented perpendicular to the coronal plane; the angle between this plane and the HF was measured, following a modified approach based on previous definitions of maxillary height [17].

### 2.5. Statistical Analysis

All statistical analyses were performed using SPSS software (version 26.0; IBM Corp., Armonk, NY, USA), with the level of significance set at α = 0.05.

For each subject, three-dimensional skeletal measurements were obtained at three time points (T0, T1, and T2), including bilateral values for all parameters. Given the paired and repeated-measures nature of the study design, statistical analyses focused on within-subject comparisons over time and between sides.

Data distribution was assessed using the Shapiro–Wilk test. Normality of paired differences (for paired comparisons) and residuals (for repeated-measures analyses) was examined to verify the assumptions of parametric testing. As the majority of variables met these assumptions and parametric methods are considered robust to moderate deviations from normality in repeated-measures designs, parametric tests were applied throughout the analysis. Accordingly, all continuous variables are presented as mean ± standard deviation (SD).

Temporal changes across the three time points (T0–T2) were evaluated separately for the long and short sides using one-way repeated-measures analysis of variance (ANOVA). When the assumption of sphericity was violated, the Greenhouse–Geisser correction was applied. Post hoc pairwise comparisons were performed with Bonferroni adjustment when a significant overall time effect was detected.

To quantify skeletal symmetry, an inter-side difference was calculated for each bilateral parameter at each time point as DTx = Tx_long_ − Tx_short_ (Tx = T0, T1, or T2). Surgical correction of asymmetry was evaluated by comparing inter-side differences between T0 and T1 (DT0 vs. DT1) using paired-sample *t*-tests. Mid-term stability of the achieved symmetry was assessed by comparing inter-side differences between T1 and T2 (DT1 vs. DT2), where an increase in D indicated a tendency toward recurrent divergence; these comparisons were also performed using paired-sample *t*-tests. This stage-based approach was adopted to address the clinically relevant questions of correction (T0–T1) and maintenance (T1–T2).

For occlusal cant analysis, an ideal value of 0° was defined as perfect symmetry. At baseline (T0), a one-sample *t*-test was applied to determine whether the mean occlusal cant angle differed significantly from 0°. Both signed values (direction of cant) and absolute values (magnitude of deviation) were compared across T0, T1, and T2 using repeated-measures ANOVA.

All tests were two-tailed.

## 3. Results

### 3.1. Baseline 3D Asymmetry (T0)

At baseline (T0), paired comparisons demonstrated significant inter-side skeletal asymmetry. Significant differences were observed in most condylar, ramus, and mandibular body parameters, whereas no significant inter-side differences were found for the anteroposterior diameter of the condyle or mandibular body height (*p* > 0.05).

In the maxilla, the long side had a significantly greater vertical height than the short side at T0 (*p* < 0.001). The occlusal plane cant angle also differed significantly from the horizontal reference at baseline (*p* < 0.001).

### 3.2. Longitudinal Three-Dimensional Skeletal Changes (T0 → T2)

Time-dependent changes in bilateral skeletal parameters were assessed using one-way repeated-measures ANOVA (Table 2).

Regarding condylar parameters, condylar height on the long side showed a significant overall time effect (*p* = 0.002), with significant reductions from T0 to T1 (*p* = 0.042) and from T0 to T2 (*p* = 0.009), whereas no significant change was observed between T1 and T2. Condylar volume and diameters on the long side did not exhibit significant time effects. On the short side, significant time effects were detected for condylar volume (*p* = 0.016) and condylar height (*p* = 0.008). Condylar volume decreased significantly from T1 to T2 (*p* = 0.023), while condylar height differed significantly between T0 and T2 (*p* = 0.018). No significant time effects were observed for condylar diameters.

For ramus and mandibular body measurements, ramus volume on the long side demonstrated a significant time effect (*p* < 0.001), increasing from T0 to T1 (*p* < 0.001) and decreasing from T1 to T2 (*p* = 0.005), with no significant difference between T0 and T2. Mandibular body length also showed a significant time effect (*p* = 0.005), characterized by a reduction from T0 to T1 (*p* = 0.013) and no further change thereafter, whereas other parameters did not show consistent post hoc differences. On the short side, significant time effects were observed for ramus volume (*p* = 0.009), mandibular body volume (*p* < 0.001), and mandibular body length (*p* < 0.001). Ramus and mandibular body volumes increased significantly from T0 to T1 and decreased or stabilized thereafter, while mandibular body length increased from T0 to T1 without further change between T1 and T2.

In the maxilla, maxillary height demonstrated significant time effects on both sides (long side: *p* < 0.001; short side: *p* = 0.030), with a significant decrease from T0 to T1 on the long side and no significant post hoc differences on the short side. The occlusal cant angle and its absolute value both showed significant time effects (both *p* < 0.001), characterized by a marked reduction from T0 to T1 and stability thereafter.

### 3.3. Changes in Inter-Side Differences During Surgical Correction and Follow-Up

Planned pairwise comparisons of inter-side (long–short) differences were performed to evaluate changes during surgical correction (T1–T0) and postoperative follow-up (T2–T1) (Table 3).

During the correction phase (T1–T0), inter-side differences in mandibular body volume, mandibular body length, and maxillary height were significantly reduced (all *p* < 0.001). A small but statistically significant reduction in inter-side difference was also observed for condylar height (*p* = 0.036). In contrast, no significant changes were detected in inter-side differences for ramus volume, condylar volume, or condylar diameters.

During the follow-up phase (T2–T1), no significant changes were observed in inter-side differences for condylar, ramus, mandibular body, or maxillary parameters (all *p* > 0.05), indicating stable inter-side relationships over the mid-term follow-up period.

## 4. Discussion

The present results demonstrate that facial asymmetry in patients with a relatively normal sagittal relationship is characterized by distinct stage-dependent changes. Pronounced skeletal asymmetry was observed at baseline, followed by a marked improvement in symmetry immediately after surgery and a stable condition with only minor remodeling during mid-term follow-up. Although the statistical analyses encompassed multiple postoperative time points, the observed changes reflect different biological phases rather than a continuous remodeling process. Accordingly, the following Discussion adopts a stage-based framework, addressing baseline structural characteristics (T0), immediate surgical effects (T0–T1), and subsequent postoperative adaptation and stability (T1–T2).

### 4.1. Structural Basis and Functional Adaptation in Facial Asymmetry

The defining feature of patients in this study was the presence of marked transverse or vertical asymmetry in the context of a relatively normal maxillomandibular sagittal relationship. Preoperative analysis revealed that most cases exhibited a skeletal pattern of “developmental predominance on the long side and developmental deficiency on the short side.” As a critical growth center of the mandible [21], the condyle on the long side exhibited markedly greater volume than that on the short side, with asymmetry predominantly expressed in the mediolateral dimension, whereas anteroposterior differences were comparatively limited. This phenomenon is consistent with observations in existing literature regarding patients with Class III mandibular asymmetry [22,23,24,25]. This finding suggests that asymmetric condylar development plays a pivotal role in the formation of mandibular deviation.

Differences in ramus height and volume were also substantial, with the long side demonstrating a clear vertical and volumetric predominance over the short side at baseline. Such vertical developmental disparity constitutes one of the major skeletal foundations for chin deviation [26]. Baek et al. reported that the magnitude of chin deviation correlates with bilateral differences in ramus height, and that excessive vertical growth of the long-side ramus can drive the chin toward the short side [17]. Although volumetric differences in the mandibular body were observed between sides, height differences were relatively small, implying a potential self-regulatory mechanism that may buffer overall deviation.

From a functional perspective, prolonged unilateral mastication and asymmetric joint loading may restrict condylar and ramus growth on the short side, while promoting compensatory overdevelopment on the long side [16,27,28,29]. Therefore, intraoperative correction should comprehensively account for the degree of asymmetry in each skeletal component to achieve both structural and functional equilibrium. This quantitative characterization of the skeletal basis provides an essential reference for subsequent analyses of bone remodeling under relatively isolated sagittal conditions.

### 4.2. Postoperative Changes in Overall Symmetry and Mechanisms of the “Marked Reversal” Phenomenon

**Figure 3:**
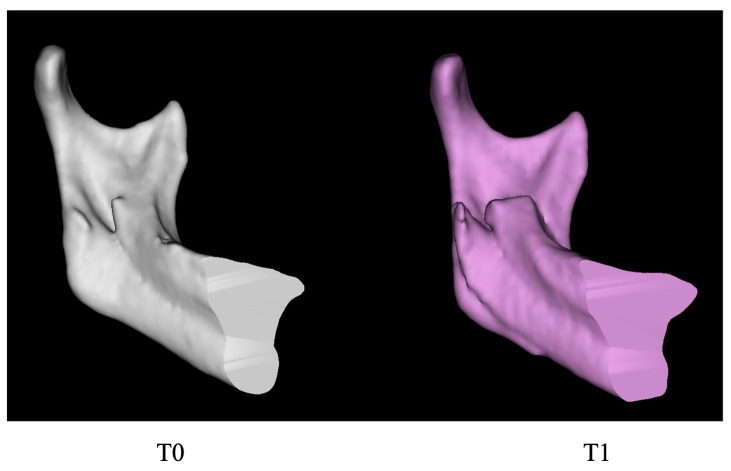
Medial aspect of the long-side mandibular ramus and body before (T0) and after (T1) surgery. Gray and purple models represent T0 and T1, respectively.

Bimaxillary orthognathic surgery markedly improved overall mandibular symmetry in the present cohort, particularly in mandibular body length and volume, where long–short-side differences were substantially reduced. However, ramus height showed minimal improvement, and the volume of the ramus exhibited only slight changes. Notably, the long-side ramus volume still increased postoperatively, which may be attributed to the necessity of repositioning the distal segment of the long-side bone, causing some mandibular body bone to move medially into the ramus, thus leading to an increase in the measured ramus volume. As illustrated in Figure 3, the preoperative (T0) and postoperative (T1) images of the medial aspect of the long-side mandibular ramus show the repositioning of the distal segment. In the T1 image, a noticeable shift in the mandibular body posteriorly is evident, with an increase in bone volume medially behind the ramus. The figure also clearly shows that the ramus height remains unchanged, further indicating that BSSRO primarily alters the position of the mandibular body, with limited effects on vertical height correction. This suggests that adjunctive contouring procedures may be necessary in certain cases [30]. In some patients, a marked reversal in mandibular body length was observed postoperatively, wherein the short side exceeded the long side. Potential mechanisms include: (1) Mild intraoperative overcorrection—to counteract soft tissue rebound and the risk of mild relapse from postoperative bone remodeling, the surgeon may intentionally advance the short side slightly beyond the ideal length intraoperatively [31], allowing it to approach symmetry over time; and (2) Passive compensation for ramus height deficiency—when the short side has insufficient ramus height that is difficult to correct intraoperatively, the distal segment may be passively positioned downward during repositioning to compensate for vertical disparity, thereby geometrically increasing mandibular body length on the short side.

These findings suggest that postoperative evaluation should prioritize 3D global harmony rather than focusing on a single linear metric. Moreover, such distinctive changes, occurring under conditions of a relatively normal sagittal relationship, should be interpreted in conjunction with mid-term remodeling patterns to provide a reliable reference for establishing normative benchmarks of postoperative bone remodeling.

### 4.3. Relationship Between Bone Remodeling and Stability

Follow-up at 6–12 months postoperatively demonstrated that postoperative skeletal remodeling had minimal impact on the stability of mandibular symmetry. Analyses of inter-side differences showed that the correction of asymmetry achieved immediately after surgery was largely maintained during follow-up, with no evidence of recurrent divergence between the long and short sides. During the same period (T1–T2), modest structural changes were observed at the level of the condyle and ramus. In particular, a small reduction in short-side condylar volume was detected, while most condylar linear dimensions remained stable. These changes were limited in magnitude and are consistent with physiological adaptation to altered postoperative loading conditions rather than pathological degeneration [32,33]. Measurements of the ramus and mandibular body likewise showed only minor variations that did not compromise overall facial symmetry.

Notably, the statistically significant findings in several condylar metrics across T0, T1, and T2 should not be interpreted as a single continuous degenerative trend. The immediate postoperative decrease in long-side condylar height (T0–T1) is more plausibly explained by intraoperative proximal-segment repositioning and spatial realignment of the condyle within the glenoid fossa, rather than true osseous loss. In contrast, the subsequent short-side condylar volume reduction observed between T1 and T2 likely reflects postoperative functional adaptation following redistribution of masticatory loading after correction of occlusal asymmetry, which is consistent with physiological remodeling. Importantly, the absence of a progressive volume loss pattern from T0 to T2 and the lack of radiographic features indicative of pathological degeneration argue against clinically relevant condylar resorption [34].

These findings indicate that, under the conditions of rigid internal fixation and standardized rehabilitation, patients in this study were able to achieve physiological readaptation without experiencing substantial structural deterioration. Modest bone remodeling reflects the skeletal adaptation process to the altered biomechanical environment and may contribute to the establishment of long-term stability [33,35,36]. Nevertheless, previous studies have reported that mild segmental repositioning may occur beyond one year postoperatively in some patients, which could indicate a risk of relapse, underscoring the need for long-term monitoring in cases of severe facial asymmetry [37,38,39,40]. Combined with the quantitative data obtained in this study under a relatively normal sagittal relationship, these mid-term remodeling characteristics provide direct evidence for formulating future reference benchmarks for postoperative bone remodeling.

### 4.4. Clinical Strategies for Maintaining Symmetry

Mid-term follow-up in the present study demonstrated that bimaxillary orthognathic surgery can substantially improve mandibular symmetry in this patient cohort and maintain a high degree of stability over 6–12 months. However, the results also indicate that symmetry maintenance is not determined by a single factor but is influenced by a combination of variables, including preoperative skeletal discrepancies, the precision of intraoperative segment repositioning and fixation, and postoperative rehabilitation and monitoring [41,42,43]. For example, residual ramus height differences were observed in some patients, and the occurrence of a marked reversal in mandibular body length on the short side suggests that mild overcorrection and passive compensation mechanisms may coexist. Furthermore, although the extent of bone remodeling in the condyle and ramus was small, their spatial position and loading environment may still exert a potential influence on long-term stability [35].

**Figure 4:**
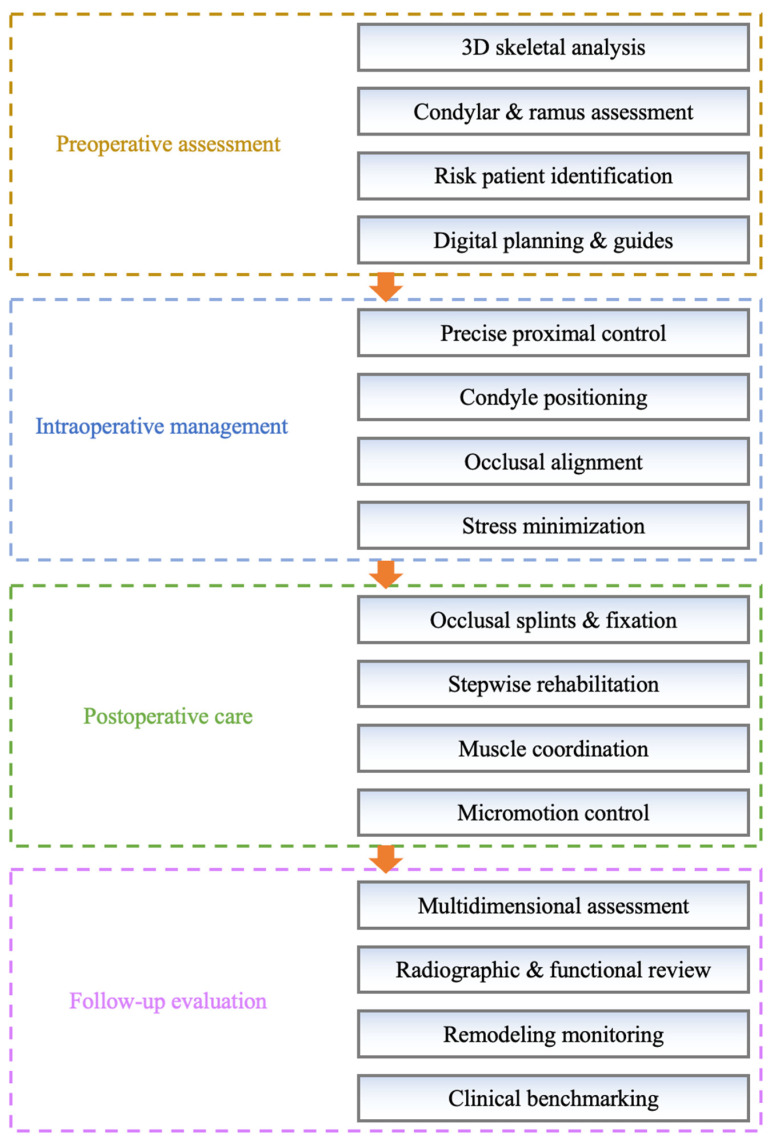
Schematic illustration of perioperative optimization strategies.

Based on these findings, the long-term maintenance of skeletal symmetry largely depends on controllable clinical factors throughout the perioperative process (Figure 4). Preoperatively, accurate evaluation of skeletal discrepancies—particularly condylar volume and ramus height differences—should be emphasized to identify high-risk patients and to formulate individualized repositioning plans with the assistance of digital simulation and surgical guides when appropriate [44,45]. Intraoperatively, precise control of the proximal segment position is essential to ensure that the condyle is placed in an anatomically appropriate position while achieving stable occlusal alignment of the distal segment, thereby minimizing joint stress and unfavorable remodeling [23,44]. Postoperatively, careful management through occlusal splints, intermaxillary fixation, and stepwise functional rehabilitation can help restrict excessive micromovement and restore muscular coordination [42]. In follow-up evaluations, a comprehensive assessment that integrates radiographic symmetry, soft tissue adaptation, and functional outcomes is recommended to provide a multidimensional understanding of stability and remodeling, rather than relying solely on a single numerical indicator [46,47].

In patients with facial asymmetry and a relatively normal sagittal relationship, the quantitative mid-term (6–12 months) bone remodeling data obtained in this study can serve as a practical reference for establishing clinical remodeling benchmarks, thereby supporting postoperative follow-up, stability evaluation, and the optimization of individualized treatment plans.

### 4.5. Limitations and Future Directions

This study has several limitations. First, the sample size was relatively small and derived from a single medical center, with a concentrated surgical team and patient demographic, which may limit the generalizability of the conclusions to other regions, surgical teams, and populations. Second, the follow-up period was 6–12 months postoperatively, representing a mid-term observation. Although this timeframe reflects early and mid-term stability, it does not provide direct evidence regarding potential mild segmental positional changes beyond one year, such as delayed segmental repositioning. Future studies should extend the follow-up period to include later postoperative stages to identify possible trends in delayed structural adjustment. In addition, although high-resolution computed tomography (CT) was employed to perform 3D measurements and reduce projection errors inherent in two-dimensional imaging, the results may still be influenced by factors such as anatomical landmark identification accuracy and reconstruction threshold settings [48]. Moreover, this study did not assess the potential influence of masticatory muscle tone or temporomandibular joint functional adaptation on skeletal remodeling, particularly in relation to the habitual chewing side, where muscular hypertrophy and ramus height asymmetry may interact [28,49].

In future research, incorporating multi-time-point longitudinal follow-up (dynamic observation) and combining analyses such as soft tissue analysis or global symmetry index evaluation could provide a more intuitive depiction of structural changes in the jaws at different postoperative stages [46,47,50]. Furthermore, multi-center collaboration, expansion of sample size, and standardization of measurement protocols will enhance the applicability and clinical relevance of the findings across diverse patient populations. In addition, future studies integrating muscle activity assessment and postoperative physical therapy evaluation may further elucidate their roles in maintaining long-term anatomical and functional harmony [51,52].

## 5. Conclusions

This study demonstrates that bimaxillary orthognathic surgery in patients with facial asymmetry and a relatively normal sagittal skeletal relationship significantly improves skeletal balance and maintains mid-term stability, with only mild morphological changes that may represent physiological remodeling rather than pathological relapse.

## Figures and Tables

**Table 1:** Reference planes and anatomical landmarks utilized in the present analysis.

Landmarks and Reference Planes	Definition
Nasion (N, R/L)	The most anterior point of the nasofrontal suture in the midsagittal plane
Porion (P, R/L)	The most superior point of the external auditory meatus
Basion (Ba)	The midpoint on the anterior margin of the foramen magnum
Orbitale (Or, R/L)	The most inferior point on the inferior margin of the orbit
Menton (Me)	The most inferior point on the inferior border of the mandibular symphysis
Gonion-inferior point (Go-inf, R/L)	The most inferior point on the angle of the mandible
Gonion-posterior point (Go-post, R/L)	The most posterior point on the angle of the mandible
Condylion (Co, R/L)	The most superior point on the condyle
Mental foramen (MF, R/L)	Posterior point of the mental foramen
Lateral jugal point (J-lat, R/L)	The most inferolateral point at the junction of the mandibular ramus and body
Medial jugal point (J-med, R/L)	The most inferomedial point at the junction of the mandibular ramus and body
Frankfort horizontal plane (HF)	The horizontal plane formed by the bilateral Or and the right P
Midsagittal plane (MSP)	The plane passing through N and Ba, perpendicular to HF
Mandibular plane (MP)	The tangent to the most inferior border of the mandible

**Table 2:** Longitudinal comparisons across T0, T1, and T2 for bilateral skeletal and occlusal plane parameters using one-way repeated-measures ANOVA with post hoc analyses.

	Measurement	T0	T1	T2	*p*	Post Hoc Comparisons		
						T0 vs. T1	T1 vs. T2	T0 vs. T2
Long Side	Condyle volume (mm^3^)	2452.54 ± 589.18	2455.49 ± 575.81	2392.23 ± 587.25	0.098	—	—	—
	Condyle mediolateral diameter (mm)	20.05 ± 2.13	20.03 ± 2.15	20.08 ± 2.31	0.960	—	—	—
	Condyle anteroposterior diameter (mm)	14.45 ± 2.29	14.80 ± 2.12	14.71 ± 2.33	0.411	—	—	—
	Condyle height (mm)	21.38 ± 2.84	20.87 ± 2.95	20.44 ± 2.92	0.002	0.042	0.073	0.009
	Ramus volume (mm^3^)	13,528.21 ± 2908.44	15,232.33 ± 3745.94	14,235.61 ± 3757.23	<0.001	<0.001	0.005	0.176
	Ramus height (mm)	71.21 ± 5.84	70.99 ± 5.86	70.04 ± 5.54	0.027	0.775	0.138	0.072
	Body volume (mm^3^)	10,603.71 ± 1603.17	10,378.85 ± 1998.21	10,221.67 ± 1871.03	0.229	—	—	—
	Body length (mm)	67.42 ± 4.54	64.32 ± 5.48	64.92 ± 5.79	0.005	0.013	0.675	0.058
	Body height (mm)	25.92 ± 3.34	26.16 ± 3.10	26.18 ± 3.09	0.470	—	—	—
	Maxillary height (mm)	41.06 ± 3.04	38.10 ± 2.21	38.38 ± 2.22	<0.001	<0.001	0.476	<0.001
Short Side	Condyle volume (mm^3^)	1465.51 ± 449.79	1486.76 ± 460.95	1411.84 ± 478.38	0.016	0.856	0.023	0.276
	Condyle mediolateral diameter (mm)	18.04 ± 2.34	17.94 ± 2.55	17.84 ± 2.63	0.427	—	—	—
	Condyle anteroposterior diameter (mm)	13.91 ± 2.20	14.19 ± 2.28	13.93 ± 2.26	0.278	—	—	—
	Condyle height (mm)	16.01 ± 2.91	15.90 ± 2.97	15.49 ± 3.04	0.008	1.000	0.107	0.018
	Ramus volume (mm^3^)	11,796.35 ± 2249.33	12,771.23 ± 2386.63	11,961.69 ± 2022.50	0.009	0.046	0.016	1.000
	Ramus height (mm)	62.31 ± 6.36	63.15 ± 5.96	62.32 ± 5.53	0.135	—	—	—
	Body volume (mm^3^)	8502.65 ± 1384.53	9872.42 ± 1596.54	9621.93 ± 1626.47	<0.001	<0.001	0.463	<0.001
	Body length (mm)	64.86 ± 4.99	67.53 ± 5.47	68.00 ± 5.28	<0.001	0.002	0.485	<0.001
	Body height (mm)	26.05 ± 2.60	25.46 ± 3.12	24.98 ± 3.20	0.043	0.503	0.533	0.097
	Maxillary height (mm)	38.21 ± 2.40	39.01 ± 2.55	38.85 ± 2.44	0.030	0.085	1.000	0.219
Occlusal cant angle (°)		2.93 ± 1.72	−0.68 ± 1.11	−0.49 ± 1.09	<0.001	<0.001	1.000	<0.001
Absolute occlusal cant angle (°)		2.93 ± 1.72	1.05 ± 0.75	0.84 ± 0.83	<0.001	0.003	0.844	0.002

Values are presented as mean ± SD (n = 25). SD, standard deviation. Post hoc comparisons are reported only for parameters showing a statistically significant overall time effect.

**Table 3:** Planned paired comparisons of inter-side (long–short) differences across surgical correction (T0–T1) and postoperative stability (T1–T2).

**Panel A. Correction (T0–T1)**				
**Measurement**	**Inter-Side Difference at T0**	**Inter-Side Difference at T1**	**T0–T1**	***p***
Condyle volume (mm^3^)	987.03 ± 551.11	968.74 ± 554.74	−18.30 ± 90.10	0.318
Condyle mediolateral diameter (mm)	2.01 ± 2.67	2.09 ± 2.79	0.09 ± 0.96	0.643
Condyle anteroposterior diameter (mm)	0.53 ± 2.00	0.61 ± 2.24	0.07 ± 1.32	0.792
Condyle height (mm)	5.37 ± 2.87	4.97 ± 3.08	−0.40 ±0.90	0.036
Ramus volume (mm^3^)	1731.85 ± 1722.66	2461.09 ± 2935.68	729.24 ± 1773.97	0.050
Ramus height (mm)	8.90 ± 4.31	7.84 ± 4.20	−1.06 ± 2.61	0.053
Body volume (mm^3^)	2101.06 ± 1575.46	506.42 ± 1570.83	−1594.63 ± 1516.49	<0.001
Body length (mm)	2.56 ± 3.46	−3.21 ± 3.37	−5.77 ± 4.40	<0.001
Body height (mm)	−0.13 ± 2.25	0.70 ± 2.28	0.83 ± 2.66	0.132
Maxillary height (mm)	2.85 ± 1.67	−0.91 ± 0.99	2.85 ±1.67	<0.001
**Panel B. Stability (T1–T2)**				
**Measurement**	**Inter-Side Difference at T1**	**Inter-Side Difference at T2**	**T1–T2**	***p***
Condyle volume (mm^3^)	968.74 ± 554.74	980.39 ± 558.10	11.66 ± 133.93	0.667
Condyle mediolateral diameter (mm)	2.09 ± 2.79	2.24 ± 2.70	0.15 ±1.00	0.237
Condyle anteroposterior diameter (mm)	0.61 ± 2.24	0.79 ± 2.18	0.18 ± 1.60	0.578
Condyle height (mm)	4.97 ± 3.08	4.95 ± 3.18	−0.02 ±1.07	0.919
Ramus volume (mm^3^)	2461.09 ± 2935.68	2273.91 ± 2504.76	−187.18 ± 1122.57	0.413
Ramus height (mm)	7.84 ± 4.20	7.72 ± 4.36	−0.12 ± 2.31	0.797
Body volume (mm^3^)	506.42 ± 1570.83	599.74 ± 1456.36	93.31 ± 1104.49	0.677
Body length (mm)	−3.21 ± 3.37	−3.08 ± 3.42	0.14 ± 2.29	0.769
Body height (mm)	0.70 ± 2.28	1.14 ± 2.13	0.44 ± 1.49	0.152
Maxillary height (mm)	−0.91 ± 0.99	−0.47 ± 1.11	0.44 ± 1.10	0.056

Values are presented as mean ± SD (n = 25). *p*-values are from two-tailed tests. SD, standard deviation. Panel A summarizes changes in inter-side differences during surgical correction (T0–T1), whereas Panel B summarizes changes during postoperative follow-up (T1–T2).

## Data Availability

The data presented in this study are available on request from the corresponding author. The data are not publicly available due to ethical and patient privacy restrictions.
